# The Survival of *Haloferax mediterranei* under Stressful Conditions

**DOI:** 10.3390/microorganisms9020336

**Published:** 2021-02-08

**Authors:** Laura Matarredona, Mónica Camacho, Basilio Zafrilla, Gloria Bravo-Barrales, Julia Esclapez, María-José Bonete

**Affiliations:** 1 Agrochemistry and Biochemistry Department, Biochemistry and Molecular Biology Area, Faculty of Science, University of Alicante, Ap 99, 03080 Alicante, Spain; lauramata1996@gmail.com (L.M.); camacho@ua.es (M.C.); basilio.zafrilla@ua.es (B.Z.); julia.esclapez@ua.es (J.E.); 2 Biotecnología Arauco SpA, Arauco 4210000, Chile; gloriabravobarrales@gmail.com

**Keywords:** *Haloferax*, stress, growth rate, doubling time, metals, *Archaea*

## Abstract

Haloarchaea can survive and thrive under exposure to a wide range of extreme environmental factors, which represents a potential interest to biotechnology. Growth responses to different stressful conditions were examined in the haloarchaeon *Haloferax mediterranei* R4. It has been demonstrated that this halophilic archaeon is able to grow between 10 and 32.5% (*w/v*) of sea water, at 32–52 °C, although it is expected to grow in temperatures lower than 32 °C, and between 5.75 and 8.75 of pH. Moreover, it can also grow under high metal concentrations (nickel, lithium, cobalt, arsenic), which are toxic to most living beings, making it a promising candidate for future biotechnological purposes and industrial applications. Inductively Coupled Plasma Mass Spectrometry (ICP-MS) analysis quantified the intracellular ion concentrations of these four metals in *Hfx. mediterranei*, concluding that this haloarchaeon can accumulate Li^+^, Co^2+^, As^5+^, and Ni^2+^ within the cell. This paper is the first report on *Hfx. mediterranei* in which multiple stress conditions have been studied to explore the mechanism of stress resistance. It constitutes the most detailed study in Haloarchaea, and, as a consequence, new biotechnological and industrial applications have emerged.

## 1. Introduction

Over the years, environmental stress has impacted all three domains of life, causing a devasting impact on growth. Microbial communities have evolved and survived through exposure to stressful conditions such as low/high solar radiation, extreme temperatures, oxidative stress, pH variations, salinity changes, heavy metals, or desiccation hydration cycles [1,2]. To survive in these conditions, organisms must adapt by shifting away from their optimum growth conditions. During a stress response, cells change from a growth state to a survival-state to return to homeostasis and avoid damage; consequently, the growth rate observed by optical density decreases.

In the last decades, extremophilic microorganisms have gained increasing attention in biotechnological applications as well as in bioremediation processes due to their unique metabolic capabilities, the production of stable enzymes under extremely hostile conditions, and unique biomaterials and/or secondary metabolites. Thermophilic and acidophilic microorganisms have been used extensively in valuable industrial applications such as biomining, industrial pollutant treatments, or as a source of enzymes. However, halophiles are under-represented in biotechnology [3,4,5]. At the time of writing this article, a small number of analyses have been carried out to study hypersaline environments, despite being distributed in a relatively large area around the world [6]; therefore, their knowledge is highly limited. Determining the in vivo behavior of a haloarchaeon and its gene expression in response to environmental variations is vital to understand how it adapts and which transcriptional changes occur to survive the ecosystem dynamics in which it is found. In the last decade, most research has pointed out that *Haloferax mediterranei* is among the best-known halophilic microorganisms belonging to the *Archaea* domain, and it has awakened the interest of many scientists. It can be used as a model organism for studying tolerance to environmental stress agents because its comprehensive knowledge in terms of biochemistry and molecular biology are widely known [7]. *Hfx. mediterranei* has several remarkable characteristics: it grows on defined media or complex media and is able to use a wide variety of carbon sources [8,9]; it has better growth rates than other known members of the *Halobacteriaceae* family [10,11]; it has a wide range of salt tolerance [12]; and its growth can be aerobic or anaerobic using different organic and inorganic sources [13]. Thanks to all these advantages, it is interesting to expand the knowledge on this organism and to find biotechnological and industrial interest applications because of its capacity to produce secondary metabolites [14,15]; environmental interest is also relevant including bioremediation of saline wastewaters generated from chemical, pharmaceutical, agricultural, and aquicultural industries [16,17,18]. Moreover, previous studies have shown that this haloarchaeon could be an attractive microorganism in terms of bioremediation as it can grow in areas with high salinity, nitrate, and nitrite to repair the damage caused by the excessive use of fertilizers used in agriculture [19].

A growing interest in climate change has emerged in recent years, and microorganisms have started to be affected due to an increase in extreme changes caused by global warming. In the case of hypersaline environments, which are often contaminated due to urbanization, industrialization, or mining, the increasing evaporation of water due to the high temperatures is causing a noticeable increase in heavy metals to toxic concentrations in these habitats. Under these conditions, most organisms are not able to deal with these rapid changes because they need optimal conditions to maintain their growth, while some microorganisms like extremophiles can tolerate and adapt to these extreme situations [20]. Therefore, understanding how *Hfx. mediterranei* adapts in nature is an essential first step to improve resiliency to climate variability.

The Mediterranean area, where *Hfx. mediterranei* was first isolated, is considered as extremely vulnerable to global warming and future climate extreme scenarios [21,22]. This region is described as a hotspot due to the changes in the mean and seasonal variability of temperature and precipitation [23]. As part of climate change, this haloarchaeon can subsist against extreme climatic events such as hot or cold individual days, sudden rains that increase the volume of water present in the saltern ponds (decreasing the salinity percentage), nutrient shortages, or the wide variability in ultraviolet radiation. All these intense stresses cause considerable harm to microbial communities. Most researchers working in the area of climate change have supported the claim that anthropogenic activities are causing significant effects on climate forcing [24] and the environment, which endanger the life of plants and animals, and that this plays a substantial role in the destruction of much of the biodiversity and natural ecosystems [25,26]. In contrast, the effect of climate change is poorly studied in microorganisms such as bacteria and archaea despite being one of the strong supporters of the biosphere [27]. 

To uncover more about the archaeal stress response, this article provides an overview of the critical factors influencing the growth of the model haloarchaeon *Hfx. mediterranei* under different stress conditions. To date, there is scarce information on the in vitro analyses of this haloarchaeon in response to different stress conditions. Therefore, this is the first study to give experimental evidence regarding the tolerance of this microorganism to the tested stress conditions. Interactions among microorganisms in saline/hypersaline ecosystems can be significantly influenced by the shifts in temperature, O_2_ concentration, salinity changes, toxic and heavy metals, pH values, and nutrient supply due to the ongoing global climate change. The present work will advance the study of the *Archaea* domain and the field of microbial ecology and environmental interest including bioremediation. 

## 2. Materials and Methods

### 2.1. Strain and Culture Media

*Hfx. mediterranei* strain R4 (ATCC 33500^T^) was grown at 42 °C in complex media (Hm-CM) containing a 20% (*w/v*) mixture of inorganic salts [9] and 0.5% (*w/v*) yeast extract per litter. The pH of the culture medium was adjusted to 7.3 with NaOH. 

*Hfx. mediterranei* defined medium (Hm-DM) contained a concentration of 20% (*w/v*) sea water and 20 mM NH_4_Cl (pH 7.3). After autoclaving and cooling, it was supplemented with 50 mM MOPS (3-(N-morpholino)propane sulfonic acid), pH 7.3, 0.03 mM FeCl_3_, 7.5 mM CaCl_2_, 27.75 mM glucose, and 1 mM KH_2_PO_4_ per liter. All microbial cultures were incubated aerobically at 42 °C with shaking (220 rpm) unless otherwise stated. Cell growth was quantified by measuring the optical density at 600 nm wavelength (OD_600_) at regular intervals (6–10 h depending on the culture’s growth rate of the culture) until reaching the stationary phase. 

### 2.2. Stress Conditions

The current investigation involved sampling and analyzing five different stress conditions: salinity, temperature, pH, H_2_O_2_, and metal stress. Hm-DM was used as basal culture media in all five experiments, varying in each one only by one parameter (sea water concentration, temperature, pH, H_2_O_2_, concentration, metal concentration). 

To analyze the effect of salt concentration on *Hfx. mediterranei* growth, cells grown in Hm-DM with a range of sea water (SW) between 10 and 32.5% (10, 12.5, 15, 17.5, 20, 22.5, 25, 27.5, 30, and 32.5% SW) without modifying the ion ratios were tested. To induce temperature stress, growth was assessed at a temperature range of 32–52 °C (32, 37, 42, 47, and 52 °C) in Hm-DM. To study pH stress in *Hfx. mediterranei*, the pH of each culture medium (Hm-DM) was adjusted differently, varying in a range between 5.75 and 8.75 (pH values were 5.75, 6.0, 6.25, 6.5, 6.75, 7.0, 7.25, 7.5, 7.75, 8.0, 8.25, 8.5, and 8.75). Oxidative stress was induced by adding H_2_O_2_ ranging from 2 to 14 mM (2, 4, 6, 8 10, 12, and 14 mM) to cultures of *Hfx. mediterranei* grown in Hm-DM at OD_600_ of 0.8 (mid-exponential phase), and the cells were cultivated for another 48–60 h. For metal resistance analysis, four metals that are toxic to most living beings at high concentrations were tested: nickel (Ni^2+^) 0.4–1.6 mM (0.4, 0.8, 1.2, and 1.6 mM); arsenic (As^5+^) 2–10 mM (2,4, 6, 8, and 10 mM); cobalt (Co^2+^) 0.2–1.2 mM (0.2, 0.4, 0.6, 0.8, 1.0, and 1.2 mM); and lithium (Li^+^) 0.5–500 mM (0.5, 2, 5, 20, 50, 250, and 500 mM). Metal salts used in the study were NiSO_4_, Na_2_HAsO_4_, CoCl_2_, and LiCl. 

Three independent biological replicates were performed for each condition to ensure reproducibility, and a control condition (Hm-DM with 20% SW, pH 7.3, 42 °C) was maintained. In the pH stress analysis, the pH of the cultures was measured daily to check the stability during growth. The starting OD_600_ of all the cultures was set to 0.02, and the lag phase, log phase, and stationary phase were statistically analyzed and compared among groups.

### 2.3. Specific Growth Rate and Doubling Time

Specific growth rate (*µ*) and doubling time (*d.t.*) were calculated by using the following equations:

(1) $$\mu =\frac{\ln X-\ln Xo}{t-to}$$

(2) $$d.t.=\frac{\ln2}{\mu }$$

*X* and *Xo* represent cell concentration at the beginning of the exponential phase and after a specific time interval, respectively.

### 2.4. Statistical Analysis

Statistical analysis was performed using GraphPad Prism (Version 8). All values in the figures are expressed as the mean of three replicates ± the standard deviation. Brown Forsythe and Welch ANOVA tests with Dunnett T3 multiple comparison tests were used to determine whether there were any statistical differences between each stress condition’s mean with the mean of the control. In all of the analyses, a value of *p* < 0.05 was considered significant.

### 2.5. Sample Treatment for Inductively Coupled Plasma Mass Spectrometry (ICP-MS)

The intracellular concentrations of the four metals’ ions (Co^2+^, Li^+^, Ni^2+^, and As^5+^) were analyzed using ICP-MS. *Hfx. mediterranei* was grown in Hm-DM at selected concentrations (Co^2+^ 0.2 and 1.2 mM, As^5+^ 4 mM, Ni^2+^ 0.5 and 1.2 mM, Li^+^ 12, 50, 250, and 500 mM). Briefly, cells were cultured in Hm-DM until the stationary phase was reached. The cells were pelleted by centrifugation at 10,000× *g* for 10 min, and the media were removed. Several washes with 20% sterile sea water were performed. Cell samples were subjected to acidic mineralization to oxidize the organic matter, solubilize all metals, and simplify the matrix. The samples were weighed on an analytical balance and placed in acid-washed 15 mL quartz digestion vials with Teflon-lined caps, to which 4 mL of HNO_3_ and 1 mL of H_2_O were added. The instrument used to perform the mineralization was the Ultrawave digestor from Milestone. The microwave program consisted of a ramp to 100 °C in 5 min, 15 min from 100 °C to 170 °C, 10 min from 170 °C to 240 °C, and a holding time of 15 min at 240 °C. Once the samples were mineralized, sample volumes were brought to 15 mL with Milli-Q water. A control cell extract (*Hfx. mediterranei* grown in Hm-DM without any metals) was treated identically and assayed.

### 2.6. Quantification of Metal Content by ICP-MS

The selected elements Co^2+^, Li^+^, Ni^2+^, and As^5+^, were determined by ICP-MS in the Research Technical Services of Alicante University. Different concentration solutions of PlasmaCal SCP33MS, SCP SCIENCE were prepared in 1% nitric acid and analyzed as calibration standards to quantify the concentration of the mentioned elements using an Agilent 8800 inductively coupled plasma mass spectrometer triple quadrupole (ICP-MS QQQ, Agilent Technologies, Las Rozas, Madrid, Spain) equipped with a micromist concentric nebulizer (Meinhard, MicroMist, Meinhard), a Peltier cooled double-pass spray chamber, standard torch, and autosampler. Data analysis was performed using the Mass Hunter workstation for ICP-MS QQQ software (Agilent Technologies, Las Rozas, Madrid, Spain) to determine the total concentration of elements in sample solutions. Two tune modes were used sequentially to ensure proper ionization and interference removal. The internal standard was also used to correct signal drift.

## 3. Results and Discussion

### 3.1. Salinity Stress

**Figure 1:**
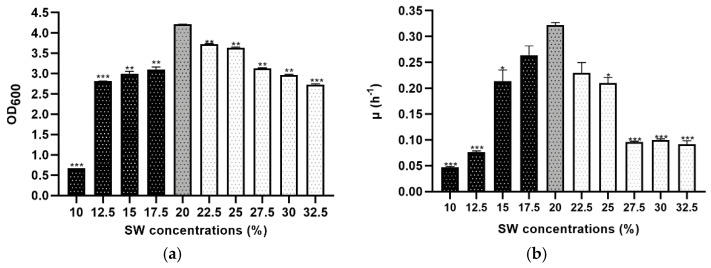
Effect of salinity stress on the growth profile of *Hfx. mediterranei* R4 grown in Hm-DM. Data are based on three independent cultures. (**a**) Maximum OD_600_ in stationary phase (reached at different times). (**b**) Average growth rate. Plotted values are the mean of triplicate measurements, and error bars represent ± SD. * *p* ≤ 0.05; ** *p* ≤ 0.01; *** *p* ≤ 0.001. In grey, the control; in black, lower concentrations of SW%; in white, higher concentrations of SW%.

A widespread form of stress observed in the Mediterranean area involves a change in external osmolarity due to extended periods of rain or drought. It was determined that members of the Haloarchaea class require a salt concentration of at least 10% (*w/v*) for growth and can survive up to 35% (*w/v*) in their natural environment, showing a significant vulnerability when the external salinity is decreased [8,9,28]. In this study, *Hfx. mediterranei* strain R4 could grow optimally in Hm-DM with a salt concentration between 10 and 32.5% (*w/v*). This suggests that its osmoregulatory responses are efficient to maintain positive growth over a wide range of salinity. Therefore, to analyze if the salinity stress substantially impacted cell growth, the three phases (lag phase, log phase, and stationary phase) of the sigmoid function were statistically analyzed and compared among groups, where the most significant were found in the stationary phase (Figure S1). Hence, mean values of optical density at 600 nm of the stationary phases are shown in Figure 1a, highlighting that not all the cultures reached this phase at the same time. There was a significant difference in mean values across almost all the conditions (except 17.5 and 22.5% (*w/v*)) and the control. Under 10% (*w/v*), no growth was detected under the assayed conditions, and a delayed growth was observed at low NaCl concentrations. Though it tolerated up to 10% of salt concentration, the growth was considerably less than the 20% control, and OD_600_ values did not reach above 0.7 (Figure 1a). Over a wide range of salt concentrations, from 27.5 to 32.5% (*w/v*), the growth curves were almost identical, and growth rates were relatively constant (Figure 1b). This result indicates that this microorganism has the necessary osmoadaptation for dealing with saturating salt conditions; in fact, good growth still occurs.

The specific growth rate and doubling time were calculated in all salinity stress conditions. The control (20%) reached the best growth rate value compared to the other salinity values, achieving 0.322 h^−1^ (Figure 1b). Value 0.322 h^−1^ was compared with an assay reported by Rodríguez-Valera, who concluded that the optimum growth rate between 10 to 35% was 20% with a value of 0.12 h^−1^ [29]. The value of 0.12 h^−1^ was worse than that of 0.322 h^−1^ because the growing conditions were not the same, despite containing the same amount of inorganic salts. In the experiment performed by Rodríguez-Valera, cultures were incubated at 38 °C, the pH was adjusted to 7.0, and the specific growth rate was calculated in a different way, taking into account all the growing curves; in our work, only the exponential phase data were used.

Table 1 represents all doubling time values, showing significant differences in almost all the cultures. The medium composition and the temperature influenced all these results.

The effect of salinity concentration on the growth of *Haloarcula marismortui* RR12 was also studied, and the results showed that the optimal growth of this haloarchaeon was reached at 20% SW [16] as *Hfx. mediterranei*. However, the comparison of growth curves between both halophilic microorganisms showed differences since *Hfx. mediterranei* reached higher DO_600_ values independently of SW concentration, except in 10% SW. Moreover, the generation times of *Hfx. mediterranei* cultures (Table 1) were lower than those of *Hal. marismortui* not only at a low salt concentration (10%), but also at high concentrations (30%). These characteristics make *Hfx. mediterranei* a promising microorganism for biotechnological applications compared to other halophiles because it can generate more biomass in a shorter time.

Deciphering the salt tolerance of *Hfx. mediterranei* will contribute to better understanding resistance and defense against toxicity at a molecular level. The high salt concentrations that this haloarchaeon can tolerate are lethal to most living organisms. 

### 3.2. pH Stress

Not only are Haloarchaea exposed to salinity changes, but they are also predisposed to changes in pH. In this study, *Hfx. mediterranei* was exposed to several different pH values, ranging from the lower (acidic pH) to upper (alkaline pH) limits for the microorganism. Apart from the composition of the culture media (Hm-DM), pH is another important requirement for optimum growth. This halophilic microorganism grows optimally or very well at neutral pH values of 7.2–7.4. In this study, pH values ranging from 5.75 to 8.75 were tested; this pH range was within the ranges previously reported for other species from the same genera [30,31]. During growth, the pH was measured to verify its stability. At pH below 5.75 and above 8.75, *Hfx. mediterranei* was unable to survive and grow. 

**Figure 2:**
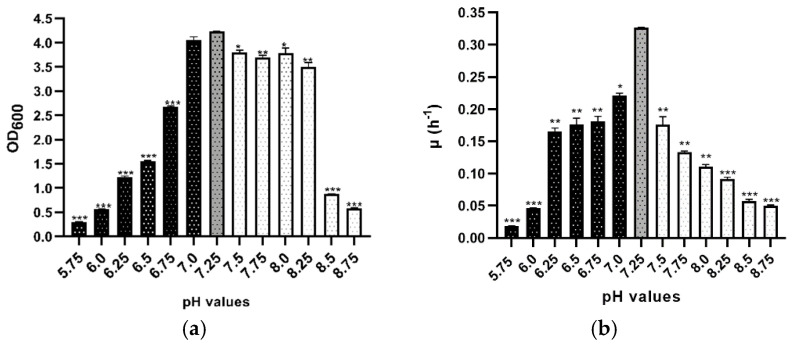
Effect of pH stress on the growth profile of *Hfx. mediterranei* R4 grown in Hm-DM. Data are based on three independent cultures. (**a**) Maximum OD_600_ in stationary phase (reached at different times). (**b**) Average growth rate. Plotted values are the mean of triplicate measurements, and error bars represent ± SD. * *p* ≤ 0.05; ** *p* ≤ 0.01; *** *p* ≤ 0.001. In grey, the control; in black, acidic pH; in white, alkaline pH.

Moreover, OD_600_ monitoring indicated a strong impact of low pH stress on cell growth, whereas high pH values caused relatively minor changes; thus, *Hfx. mediterranei* tolerates high pH better than low pH (Figure 2a and Figure S2). To analyze the impact of changing pH values, the maximum optical density reached at different times by cultures was statistically analyzed and compared. A strong impact was detected in the stationary phases when the pH was far away from the optimal pH, particularly in 5.75, 6.0, 8.5, and 8.75 (Figure 2a). 

As can be seen in Figure 2b and Table 2, the growth kinetics were also analyzed. The control (pH 7.25) reached the best results, underscoring the importance of maintaining the pH value. Furthermore, even though pH 7.0 showed no significant differences in the maximum optical density achieved by the culture (Figure 2a), significant differences were detected in terms of growth rate (Figure 2b) and doubling time (Table 2). At lower pH values, cells grew much slower than at high values of pH. In all tested conditions, data for each pH value were significant among the groups and the control (*p* ≤ 0.05), as shown in Figure 2b and Table 2. These results show that although the growth was slower at extreme acidic or alkaline pH, *Hfx. mediterranei* R4 can grow over a wide pH range, and the study of its adaptation strategies may provide interesting insights into the physiology and survival mechanisms of Haloarchaea.

### 3.3. Temperature Stress

Focusing on the response to changes in temperature, *Hfx. mediterranei* was exposed to different temperature values to analyze its broad tolerance. In previous studies, the lower-temperature limit tested for growth was 12 °C and the upper-temperature limit tested was 55 °C [10]. In this study, *Hfx. mediterranei* was able to grow in Hm-DM in a temperature range of 32–52 °C. However, its optimum growth occurred at 42 °C. Measurable growth was observed down to a temperature of 32 °C, though growth was very slow. The growth temperature optimum and minimum for *Hfx. mediterranei* are typical of most Haloarchaea [10,32]. Halophiles have a slight thermophilic character due to the hot weather seasonal temperatures of the environment in which they live.

**Figure 3:**
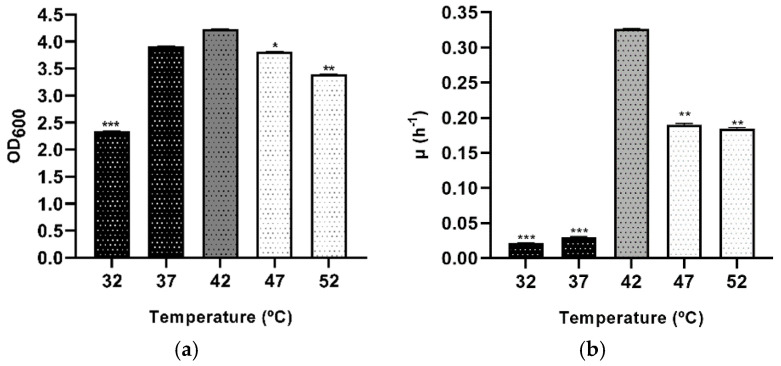
Effect of temperature stress on the growth profile of *Hfx. mediterranei* R4 grown in Hm-DM. Data are based on three independent cultures. (**a**) Maximum OD_600_ in stationary phase (reached at different times). (**b**) Average growth rate. Plotted values are the mean of triplicate measurements, and error bars represent ± SD. * *p* ≤ 0.05; ** *p* ≤ 0.01; *** *p* ≤ 0.001. In grey, the control; in black, lower temperatures; in white, higher temperatures.

As in the other previous stress conditions, all the cultures’ stationary phases were statistically compared and analyzed, highlighting that this stationary phase was the maximum growth reached at different times by cultures. There was a significant difference detected in mean values across 32, 47, 52 °C, and the control (42 °C) (Figure 3a); therefore, the growth curves of cultures that showed a difference were displayed (Figure S3). Furthermore, focusing on growth kinetics, changes in the specific growth rates and doubling times of the groups are shown in Figure 3b and Table 3. These values were affected by changes in temperature. As shown in Figure 3b, at temperature values lower than the optimal (black bars), cells grew much lower than at high values (white bars). Moreover, the lowest growth values were found at 32 and 37 °C with 0.02 and 0.03 h^−1^, respectively.

In other previous studies, the minimum doubling time of *Hfx. mediterranei* reported was as low as 1.20 h in complex medium at 47–54 °C and 2.07 h in defined medium at 43–51 °C [11]. These results are similar to the ones obtained in this research. As shown in Table 3, the control culture reached the lowest doubling time of 2.13 h. This ability to survive in high temperatures makes this haloarchaeon a potential microorganism to use in biotechnological processes since its enzymes do not run the risk of denaturation.

### 3.4. Oxidative Stress

**Figure 4:**
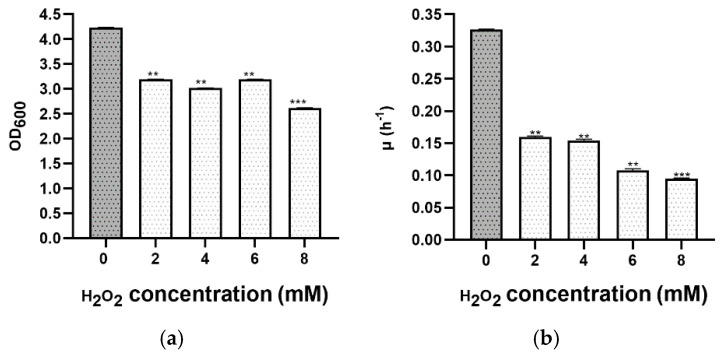
Effect of oxidative stress on the growth profile of *Hfx. mediterranei* R4 grown in Hm-DM. Data are based on three independent cultures. (**a**) Maximum OD_600_ in stationary phase (reached at different times). (**b**) Average growth rate. Plotted values are the mean of triplicate measurements, and error bars represent ± SD. ** *p* ≤ 0.01; *** *p* ≤ 0.001. In grey, the control without H_2_O_2_; in white, with H_2_O_2._

To show the resistance of *Hfx. mediterranei* to oxidative stress, this hypersaline archaeon was exposed in the exponential phase to various hydrogen peroxide concentrations ranging from 2 to 14 mM. All cultures continued to grow, exhibiting differences from the non-stressed control (same medium culture without adding peroxide). At first, the growth curves were almost identical, but a significant difference was observed in the maximum optical density reached at different times (Figure 4 and Figure S4). The effect was relatively mild after the addition of hydrogen peroxide (H_2_O_2_) between 2 and 8 mM, in contrast to 10–14 mM, which led to a considerable reduction in growth, and cultures only reached an optical density of 1.0 at 600 nm, taking into account that the hydrogen peroxide was added at 0.8. Cells could not tolerate concentrations higher than 16 mM; therefore, no significant growth was observed. Furthermore, comparing these results with other similar previous work showed that other Haloarchaea (e.g., *Hbt. salinarum* NRC-1) are highly more resistant than *Hfx. mediterranei* to oxidative stress. *Hbt. salinarum* grows in remarkably H_2_O_2_ stressful conditions and survives up to 25 mM H_2_O_2_, whereas 16 mM H_2_O_2_ is lethal to *Hfx. mediterranei* [33]. This fact could result from differences in the thickness and composition of the S-layer between both halophilic microorganisms. Therefore, all these pieces of evidence demonstrate that *Hfx. mediterranei* can grow in the presence of up to 16 mM of H_2_O_2_, but this haloarchaeon is not able to grow if the hydrogen peroxide is added from the beginning of growth.

The specific growth rate and doubling time of the exponential growth phase were determined at each concentration of H_2_O_2_ (Figure 4b and Table 4), using the data from the growth curve. As expected, the higher the peroxide concentration s, the slower the growth rate and the higher the doubling time. Similar values of cell growth were observed in cultures with 2 and 4 mM of H_2_O_2_.

### 3.5. Metal Stress

**Figure 5:**
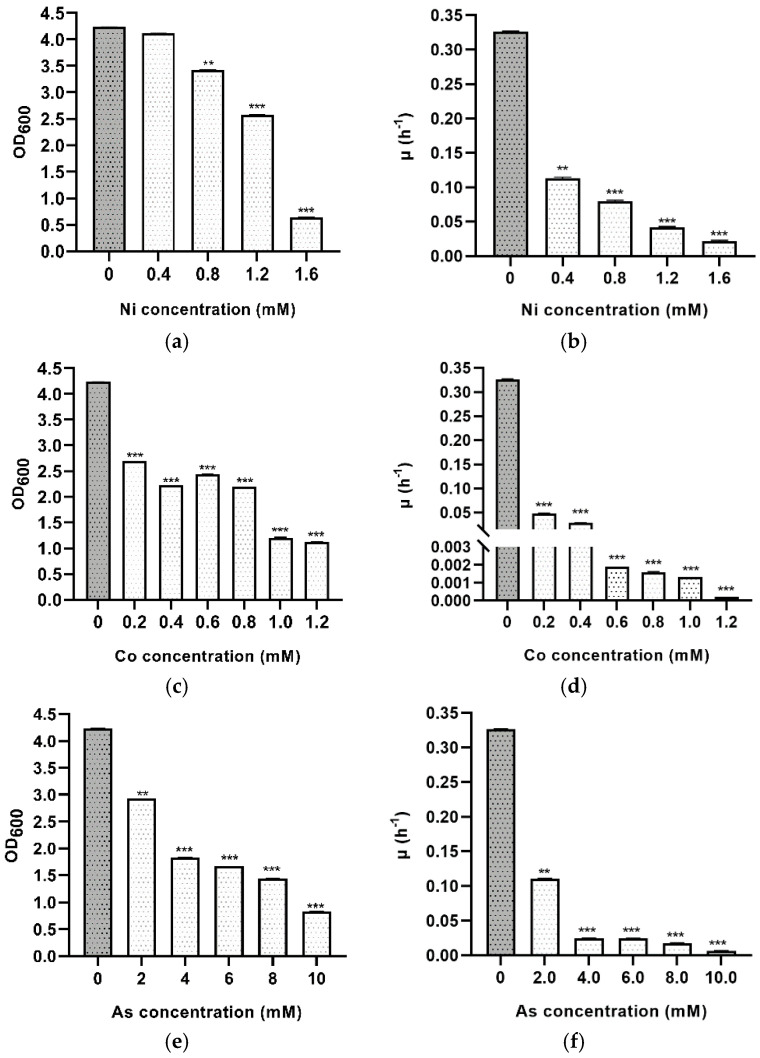

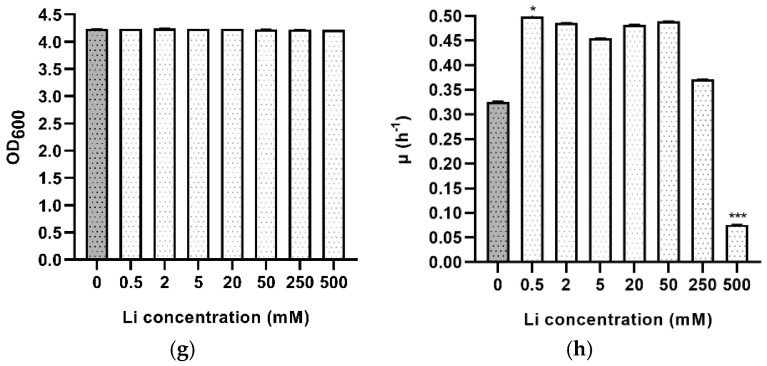
Effects of heavy metal stress on the growth profile of *Hfx. mediterranei* R4 grown in Hm-DM. Data are based on three independent cultures. (**a**) Maximum OD_600_ in stationary phase adding Ni^2+^ (reached at different times). (**b**) Average growth rate with Ni^2+^ (**c**) Maximum OD_600_ in the stationary phase adding Co^2+^ (reached at different times). (**d**) Average growth rate with Co^2+^ (**e**). Maximum OD_600_ in stationary phase adding As^5+^ (reached at different times). (**f**) Average growth rate with As^5+^. (**g**) Maximum OD_600_ in the stationary phase adding Li^+^ (reached at the same times). (**h**) Average growth rate with Li^+^. Plotted values are the mean of triplicate measurements, and error bars represent ± SD. * *p* ≤ 0.05; ** *p* ≤ 0.01; *** *p* ≤ 0.001. In grey, control without toxic metals; in white, with toxic metals.

Hypersaline environments or contaminated sea waters are natural environments where few microorganisms can survive due to the high salinity. These places are also generally contaminated with heavy metals due to urban and industrial discharge. As haloarchaea can thrive in such extreme environments, they are of great importance due to their capacity to deal with metals. Most metals that are toxic to microorganisms at high concentrations are released due to anthropogenic activities such as from agricultural waste or industrial disposals. The importance of microorganisms with metabolic capabilities to tolerate salt and metals simultaneously is increasing. Hence, the current study focuses on the tolerance of *Hfx. mediterranei* toward nickel, cobalt, arsenic, and lithium using different concentrations of metal salts. At the time of writing this article, few essays have been carried out to study the natural susceptibility of Halobacteria to heavy metals. To analyze the sensibility of *Hfx. mediterranei* to these metals, the maximum optical density reached at different times by the cultures were statistically analyzed and compared among groups and the control (non-stressed) (Figure 5). The behavior of this haloarchaeon was not homogeneous concerning all tested metals. From a general point of view, *Hfx. mediterranei* can tolerate Co^2+^, As^5+^, Li^+^, and Ni^2+^, but in different ways. The optical density was monitored, showing a strong impact of Co^2+^ stress on cell growth, whereas the addition of Ni^2+^ caused relatively minor changes. For Ni, *Hfx. mediterranei* could grow up to 1.6 mM, reaching a maximum OD_600_ of 0.65. Above 1.6 mM, no growth was detected. Therefore, the minimum inhibitory concentration of nickel tested against *Hfx. mediterranei* was 1.6 mM. There was a significant difference in the mean values of the stationary phases across almost all conditions (except 0.4 mM) and the control (Figure 5a and Figure S5). *Hfx. mediterranei* showed consistent growth at all nickel concentrations, with a gradual reduction in growth with increasing nickel concentration. Other members from the same genus such as *Hbt. salinarum*, *Hfx. gibbonsii*, *Hfx. hispanicum*, *Hfx. volcanii*, or *Hfx. vallismortis* can grow up to similar concentrations [34,35,36]; however, *Haloarcula* sp. can only grow up to 0.1 mM of Ni [37]. Environmental pollution of hypersaline environments with nickel and other toxic metals is due to anthropogenic activities such as industrialization [38] since these habitats act as sinks for these metals. Although more work is needed, these data are an excellent start point to use *Hfx. mediterranei* in bioremediation. Moreover, Ni^2+^ is industrially relevant since nickel–hydrogen batteries for large-scale energy storage have been acclaimed as an advanced power source [39].

Regarding the addition of Co^2+^, *Hfx. mediterranei* R4 could not grow in concentrations of cobalt higher than 1.2 mM. When cells were exposed to 1.0 and 1.2 mM, the stationary phase was reached at an optical density around 1, showing high mortality. Independent of Co^2+^ concentration, the OD_600_ value decreased with its presence (Figure 5c and Figure S6). In all tested concentrations with the addition of Co^2+^, the growth curves showed an initial long lag phase indicating difficulties to start growing. These results are similar to the ones obtained in *Hfx. mediterranei* by Nieto, who concluded that the lowest concentration of Co^2+^, which prevented growth, was 1 mM [37]. Other microorganisms such as *Hfx. gibbonsii*, *Hfx. hispanicum*, *Hfx. volcanii*, *Hfx. vallismortis*, and *Haloarcula* sp. also grow up to 1 mM; however, *Hbt. halobium* only grows up to 0.5 mM [37]. As cobalt has been found in industrial wastewaters, its removal is essential due to its serious health effects and environmental hazard. In terms of future applications, finding out the biosorption capacity of *Hfx. mediterranei* for bioremediation of Co^2+^ from contaminated water in natural environments would be one crucial biotechnological application. In addition to being relevant in bioremediation, it is also useful in the mining industry to produce cobalt [40].

Arsenic ions are very toxic to most microorganisms, explaining the more prolonged lag phase experienced by *Hfx. mediterranei*. However, this haloarchaeon is not the only one that can grow in the presence of arsenic. Previous work showed that 20 mM is the lowest metal concentration that prevented the growth of *Hbt. salinarum* and *Hfx. volcanii* [37]. In *Hfx. mediterranei*, all cultures had a lag phase of 48 h before starting to grow, and significant differences were detected in mean values across all tested concentrations (Figure 5e and Figure S7). Arsenate concentrations higher than 10 mM were not tested because the resulting optical density was imprecise due to the medium’s turbidity. However, a previous assay reported that *Hfx. mediterranei* could withstand 20 mM of arsenate [37]. Future assays should be based on studying the mechanism of arsenic resistance in *Hfx. mediterranei* because little work has been reported on this subject, and new industrial and biotechnological applications may appear. 

As lithium is widely distributed worldwide and has several industrial applications, it is important to know if *Hfx. mediterranei* can grow in its presence. It is remarkable that this is the first study on the tolerance of lithium that has been carried out in Haloarchaea. In Li^+^ tolerance assays, unexpected results were obtained. The cultures reached the same density in the stationary phase, ranging between 0.5 and 500 mM, indicating no difference from the control (Figure 5g). Therefore, *Hfx. mediterranei* R4 can grow in lithium’s presence up to 0.5 M. The overall growth pattern upon exposure to Li^+^ was not similar to the control, in fact, cultures reached a stationary phase before the control (Figure S8). The growth kinetics showed that the presence of these metals (toxic to most living beings) in the media resulted in a lower growth rate, except in Li’s case (Figure 5/Table 5). Adding Li^+^ resulted in a better growth rate and doubling time. Recently, the use of microorganisms in metal recovery has been increased; however, there is still little information about lithium’s recovery [41]. In the future, proteomic or RNA-Seq approaches, among others, would help understand the mechanisms implicated in the tolerance of *Hfx. mediterranei* to Li^+^ and try to elucidate the strategy followed by this haloarchaeon to adapt to Li^+^. Its demand has been increased exponentially in the last years due to its applications in lithium-ion rechargeable batteries [42,43]. These batteries can be used to supply power for several portable electrical devices. It has applications in batteries and is also used in many items such as the manufacture of glass and ceramics or in the treatment of psychiatric disorders. Consequently, its recovery is a crucial subject, making *Hfx. mediterranei* a great biotechnological application.

There are few studies of tolerance to Li; one of them has been carried out in *Rhodococcus* sp. A5wh. This bacterium responds to lithium stress by overexpressing of stress proteins, proteins related to tricarboxylic acid cycle (TCA), and enzymes involved in the synthesis of compounds that can be used as osmoprotectants. Glutamine synthetase was overexpressed in the presence of Li, and this enzyme catalyzes the condensation of glutamate and ammonia to glutamine, an amino acid involved in protein and purine synthesis. It can act as a carbon source to recharge the TCA [44]. In the case of *Hfx. mediterranei*, similar mechanisms to guarantee adaptation to the environment in response to Li may take place because this haloarchaeon grows faster in the presence of Li compared to the control.

### 3.6. Detection of Intracellular Metal Ion Concentration Using ICP-MS

The role of the tested metals during the stress response of *Hfx. mediterranei* was further investigated using the highly sensitive technique of ICP-MS. It was observed that Co^2+^, Ni^2+^, Li^+^, and As^5+^ were detected inside the cells in Hm-DM (Table 6). The control (Hm-DM without metals) contained neither cobalt nor arsenic (0 mg/Kg). However, nickel (0.17 mg/Kg) and lithium (0.50 mg/Kg) were present due to the sea water composition. Focusing on cobalt and nickel, increasing the metal’s concentration to the culture medium increased the intracellular ion accumulation. In the case of lithium, its intracellular concentration increased to a maximum as there were no differences between the cultures with Li 250 and 500 mM. It is demonstrated that *Hfx. mediterranei* can absorb Li^+^, Co^2+^, As^5+^, and Ni^2+^ within the cell, and future assays are needed to exploit the potential of this haloarchaeon in terms of its biotechnological applications.

## 4. Conclusions

This work was the first to catalogue how *Hfx. mediterranei* can survive and grow under different extreme or stressful environmental conditions. It has been demonstrated that *Hfx. mediterranei* can adapt to harsh conditions such as low/high temperatures, low/high salt concentrations, changes in pH, oxidative stress, and the addition of metals (Ni^2+^, Li^+^, Co^2+^, As^5+^). Adapting to extreme conditions is an important advantage of living beings, considering, on one hand, that extreme adverse conditions are increasingly frequent due to climate change and, on the other hand, the possibility of developing new and promising biotechnological processes.

To sum up, *Hfx. mediterranei* grows optimally at 20% (*w/v*) of salt, pH 7.25, and 42 °C, although it can grow in a broader range of salt (10–35% SW), pH (6.25–8.25), and temperature (32–52 °C). These results indicate the use of this halophilic microorganism in diverse and interesting areas of study such as understanding its molecular adaptations and mechanisms against these stress conditions that are lethal to most living organisms; transcriptomic and proteomic analyses of salt, pH, or temperature tolerance mechanisms; genetic engineering studies; and new biotechnological industrial applications such as the development of halotolerant enzymes and development of biochemical processes in media with high ionic strength, temperature, and acidic or basic pHs.

According to this study, *Hfx. mediterranei* can tolerate Li^+^, Co^2+^, As^5+^, and Ni^2+^ at different concentrations in defined media, although each of these elements affect the specific growth rate of the microorganism in a particular way. The behavior of this microorganism in the presence of nickel and arsenic is similar to those described in other halophiles such as *Hbt. salinarum* or *Halobacillus*. The growth kinetics were more sensitive to cobalt and arsenic than for nickel and, especially, for lithium. Strikingly, *Hfx. mediterranei* can tolerate lithium concentrations up to 500 mM. Furthermore, its growth in the presence of Li^+^ is better than in its absence, characterized by the shorter generation time. Furthermore, not only can it tolerate and grow in the presence of lithium, but it also incorporates it into its cellular interior. In light of these results, this haloarchaeaon is an excellent candidate to be used in biotechnology for the bioremediation of heavy metals in contaminated environments.

This work has shown that *Hfx. mediterranei* can respond and adapt quickly to a multitude of adverse conditions. Therefore, it can be considered as a microorganism with a polyextremophilic character that is useful to develop new biotechnological applications. Further in-depth studies are necessary to elucidate its function and structural adaptation in greater detail against stress conditions in order to exploit its potential applications. The authors hope that this article will encourage other experts to investigate stress in Haloarchaea, to expand the knowledge of their natural environments, limitations, and mechanisms, and to adapt to these environmental shifts. Details about metal resistance and the utilization of Haloarchaea have been described here for first time; however, biotransformation of metals and novel bioremediation strategies remain uncovered. Although the current situation shows that haloarchaea are under-explored at the biotechnological level and their available information is scarce compared to other microorganisms living in non-halophilic environments, this work undoubtedly entails a great advance in this field.

## Figures and Tables

**Table 1:** Doubling time for *Hfx. mediterranei* under salinity stress conditions.

%SW	d.t. (h) *	*p*-Value	Summary
10	14.8 ± 0.5	<0.001	***
12.5	9.1 ± 0.2	<0.001	***
15	3.4 ± 0.2	0.037	*
17.5	2.7 ± 0.2	0.148	ns
20	2.15 ± 0.03		Control
22.5	3.0 ± 0.3	0.138	ns
25	3.3 ± 0.2	0.037	*
27.5	7.2 ± 0.1	<0.001	***
30	6.91 ± 0.09	<0.001	***
32.5	7.75 ± 0.05	<0.001	***

ns: *p* > 0.05; * *p* ≤ 0.05; *** *p* ≤ 0.001. * *Hfx. mediterranei* growth in liquid culture was assessed by optical density at 600 nm, and doubling times during exponential phase growth were calculated as described in the Materials and Methods section.

**Table 2:** Doubling time for *Hfx. mediterranei* growth under different pH stress conditions.

pH	d.t. (h) *	*p*-Value	Summary
5.75	36.9 ± 0.3	<0.001	***
6.0	15.0 ± 0.3	<0.001	***
6.25	3.9 ± 0.1	0.006	**
6.5	4.2 ± 0.1	0.003	**
6.75	3.13 ± 0.05	0.03	**
7.0	3.8 ± 0.2	0.004	*
7.25	2.15 ± 0.03	-	Control
7.5	5.22 ± 0.09	0.001	**
7.75	6.3 ± 0.2	0.003	**
8.0	4.0 ± 0.3	0.003	**
8.25	7.6 ± 0.2	<0.001	***
8.5	12.1 ± 0.2	<0.001	***
8.75	14.0 ± 0.3	<0.001	***

ns: *p* > 0.05; * *p* ≤ 0.05; ** *p* ≤ 0.01; *** *p* ≤ 0.001. * *Hfx. mediterranei* growth in liquid culture was assessed by optical density at 600nm, and doubling times during exponential phase growth were calculated as described in the Materials and Methods section.

**Table 3:** Doubling time of *Hfx. mediterranei* growth under different temperatures.

Temperature (°C)	d.t. (h) *	*p*-Value	Summary
32	31.8 ± 0.4	<0.001	***
37	23.0 ± 0.4	<0.001	***
42	2.13 ± 0.03	-	Control
47	3.65 ± 0.04	0.004	**
52	3.75 ± 0.03	0.003	**

** *p* ≤ 0.01; *** *p* ≤ 0.001. * *Hfx. mediterranei* growth in liquid culture was assessed by optical density at 600 nm, and doubling times during the exponential phase growth were calculated as described in the Materials and Methods section.

**Table 4:** Doubling time of *Hfx. mediterranei* growth in the presence of different H_2_O_2_ concentrations to induce oxidative stress.

[H_2_O_2_] (mM)	d.t. (h) *	*p*-Value	Summary
0	2.13 ± 0.03	-	Control
2	4.34 ± 0.04	0.003	**
4	4.51± 0.08	0.003	**
6	6.4 ± 0.1	0.001	**
8	7.3 ± 0.1	<0.001	***

** *p* ≤ 0.01; *** *p* ≤ 0.001. * *Hfx. mediterranei* growth in liquid culture was assessed by optical density at 600 nm, and doubling times during the exponential phase growth were calculated as described in the Materials and Methods section.

**Table 5:** Doubling time of *Hfx. mediterranei* growth in the presence of Ni, Co, As, or Li at different concentrations.

Heavy Metal	Concentration (mM)	d.t. (h) *	*p*-Value	Summary
-	-	2.15 ± 0.03	-	Control
Nickel	0.5	6.14 ± 0.09	0.001	**
	0.8	8.7 ± 0.2	<0.001	***
	1.2	16.6 ± 0.4	<0.001	***
	1.6	31.9 ± 1.6	<0.001	***
Cobalt	0.2	14.24 ± 0.06	<0.001	***
	0.4	24.1 ± 0.2	<0.001	***
	0.6	364.81 ± 0.01	<0.001	***
	0.8	442.84 ± 0.02	<0.001	***
	1	533.19 ± 0.01	<0.001	***
	1.2	3465.73 ± 0.02	<0.001	***
Arsenic	2	6.28 ± 0.03	0.001	**
	4	28.1 ± 0.2	<0.001	***
	6	28.5 ± 0.2	<0.001	***
	8	40 ± 1	<0.001	***
	10	105 ± 2	<0.001	***
Lithium	0.5	1.390 ± 0.004	0.13	*
	2	1.426 ± 0.003	0.263	ns
	5	1.523 ± 0.001	>0.999	ns
	20	1.437 ± 0.004	0.832	ns
	50	1.416 ± 0.002	0.065	ns
	250	1.865 ± 0.002	>0.999	ns
	500	9.11 ± 0.09	<0.001	***

ns: *p* > 0.05; * *p* ≤ 0.05; ** *p* ≤ 0.01; *** *p* ≤ 0.001. * *Hfx. mediterranei* growth in liquid culture was assessed by optical density at 600 nm, and doubling times during the exponential phase growth were calculated as described in the Materials and Methods section.

**Table 6:** Detection of intracellular metal ion accumulation by inductively coupled plasma mass spectrometry (ICP-MS) in *Hfx. mediterranei* R4 cultured in Hm-DM.

Medium	Cobalt (mg/Kg)	Nickel (mg/Kg)	Lithium (mg/Kg)	Arsenic (mg/Kg)
Control	0.00 ± 0.00	0.17 ± 0.00	0.50 ± 0.00	0.00 ± 0.00
Co 0.2 mM	1.48 ± 0.01	-	-	-
Co 1.2 mM	2.41 ± 0.09	-	-	-
As 4 mM	-	-	-	0.17 ± 0.09
Ni 0.5 mM	-	0.32 ± 0.01	-	-
Ni 1.2 mM	-	1.37 ± 0.15	-	-
Li 12 mM	-	-	0.64 ± 0.01	-
Li 50 mM	-	-	0.66 ± 0.01	-
Li 250 mM	-	-	1.56 ± 0.02	-
Li 500 mM	-	-	1.55 ± 0.02	-

## Supplementary Materials

The following are available online at https://www.mdpi.com/2076-2607/9/2/336/s1, Figure S1: Growth of *Hfx. mediterranei* R4 under (●) standard culture conditions (20% SW, 20 mM NH_4_Cl, 50 mM MOPS, 0.03 mM FeCl_3_, 7.5 mM CaCl_2_, 22.75 mM glucose. The pH was adjusted to 7.3 and grown at 42 °C. No metal addition.) and under (●) salt stress conditions: (a) 10% SW; (b) 12.5% SW; (c) 15% SW; (d) 17.5% SW; (e) 22.5% SW; (f) 25% SW; (g) 27.5% SW; (h) 30% SW; (i) 32.5% SW, Figure S2: Growth of *Hfx. mediterranei* R4 under (●) standard culture conditions (20% SW, 20 mM NH_4_Cl, 50 mM MOPS, 0.03 mM FeCl_3_, 7.5 mM CaCl_2_, 22.75 mM glucose. The pH was adjusted to 7.3 and grown at 42 °C. No metal addition.) and under (●) pH stress conditions: (a) pH 5.75; (b) pH 6.0; (c) pH 6.25; (d) pH 6.5; (e) pH 6.75; (f) pH 7.0; (g) pH 7.5; (h) pH 7.75; (i) pH 8.0; (j) pH 8.25; (k) pH 8.5; (l) pH 8.75, Figure S3: Growth of *Hfx. mediterranei* R4 under (●) standard culture conditions (20% SW, 20 mM NH_4_Cl, 50 mM MOPS, 0.03 mM FeCl_3_, 7.5 mM CaCl_2_, 22.75 mM glucose. The pH was adjusted to 7.3 and grown at 42 °C. No metal addition.) and under (●) temperature stress conditions: (a) 32 °C; (b) 37 °C; (c) 47 °C; (d) 52 °C, Figure S4: Growth of *Hfx. mediterranei* R4 under (●) standard culture conditions (20% SW, 20 mM NH_4_Cl, 50 mM MOPS, 0.03 mM FeCl_3_, 7.5 mM CaCl_2_, 22.75 mM glucose. The pH was adjusted to 7.3 and grown at 42 °C. No metal addition.) and under (●) oxidative stress conditions induced by H_2_O_2_: (a) 2 mM H_2_O_2_; (b) 4 mM H_2_O_2_; (c) 6 mM H_2_O_2_; (d) 8 mM H_2_O_2_, Figure S5: Growth of *Hfx. mediterranei* R4 under (●) standard culture conditions (20% SW, 20 mM NH_4_Cl, 50 mM MOPS, 0.03 mM FeCl_3_, 7.5 mM CaCl_2_, 22.75 mM glucose. The pH was adjusted to 7.3 and grown at 42 °C. No metal addition) and under (●) metal stress conditions induced by nickel: (a) 0.4 mM Ni^2+^; (b) 0.8 mM Ni^2+^; (c) 1.2 mM Ni^2+^; (d) 1.6 mM Ni^2+^, Figure S6: Growth of *Hfx. mediterranei* R4 under (●) standard culture conditions (20% SW, 20 mM NH_4_Cl, 50 mM MOPS, 0.03 mM FeCl_3_, 7.5 mM CaCl_2_, 22.75 mM glucose. The pH was adjusted to 7.3 and grown at 42 °C. No metal addition.) and under (●) metal stress conditions induced by cobalt: (a) 0.2 mM Co^2+^; (b) 0.4 mM Co^2+^; (c) 0.6 mM Co^2+^; (d) 0.8 mM Co^2+^; (e) 1.0 mM Co^2+^; (f) 1.2 mM Co^2+^, Figure S7: Growth of Hfx. mediterranei R4 under (●) standard culture conditions (20% SW, 20 mM NH4Cl, 50 mM MOPS, 0.03 mM FeCl3, 7.5 mM CaCl2, 22.75 mM glucose. The pH was adjusted to 7.3 and grown at 42 °C. No metal addition.) and under (●) metal stress conditions induced by arsenic: (a) 2 mM As5+; (b) 4 mM As5+; (c) 6 mM As5+; (d) 8 mM As5+; (e) 10 mM As5+, Figure S8: Growth of *Hfx. mediterranei* R4 under (●) standard culture conditions (20% SW, 20 mM NH_4_Cl, 50 mM MOPS, 0.03 mM FeCl_3_, 7.5 mM CaCl_2_, 22.75 mM glucose. The pH was adjusted to 7.3 and grown at 42 °C. No metal addition.) and under (●) metal stress conditions induced by lithium: (a) 0.5 mM Li^+^; (b) 2 mM Li^+^; (c) 5 mM Li^+^; (d) 20 mM Li^+^; (e) 50 mM Li^+^; (f) 250 mM Li^+^; (g) 500 mM Li^+^.
