# Supplementary material for: The Survival of Haloferax mediterranei under Stressful Conditions
Source: Microorganisms. 2021 Feb 8;9(2):336. doi: 10.3390/microorganisms9020336 (PMC7915512; doi:10.3390/microorganisms9020336)
Supplement: Supplementary file 1 [file microorganisms-09-00336-s001.pdf]

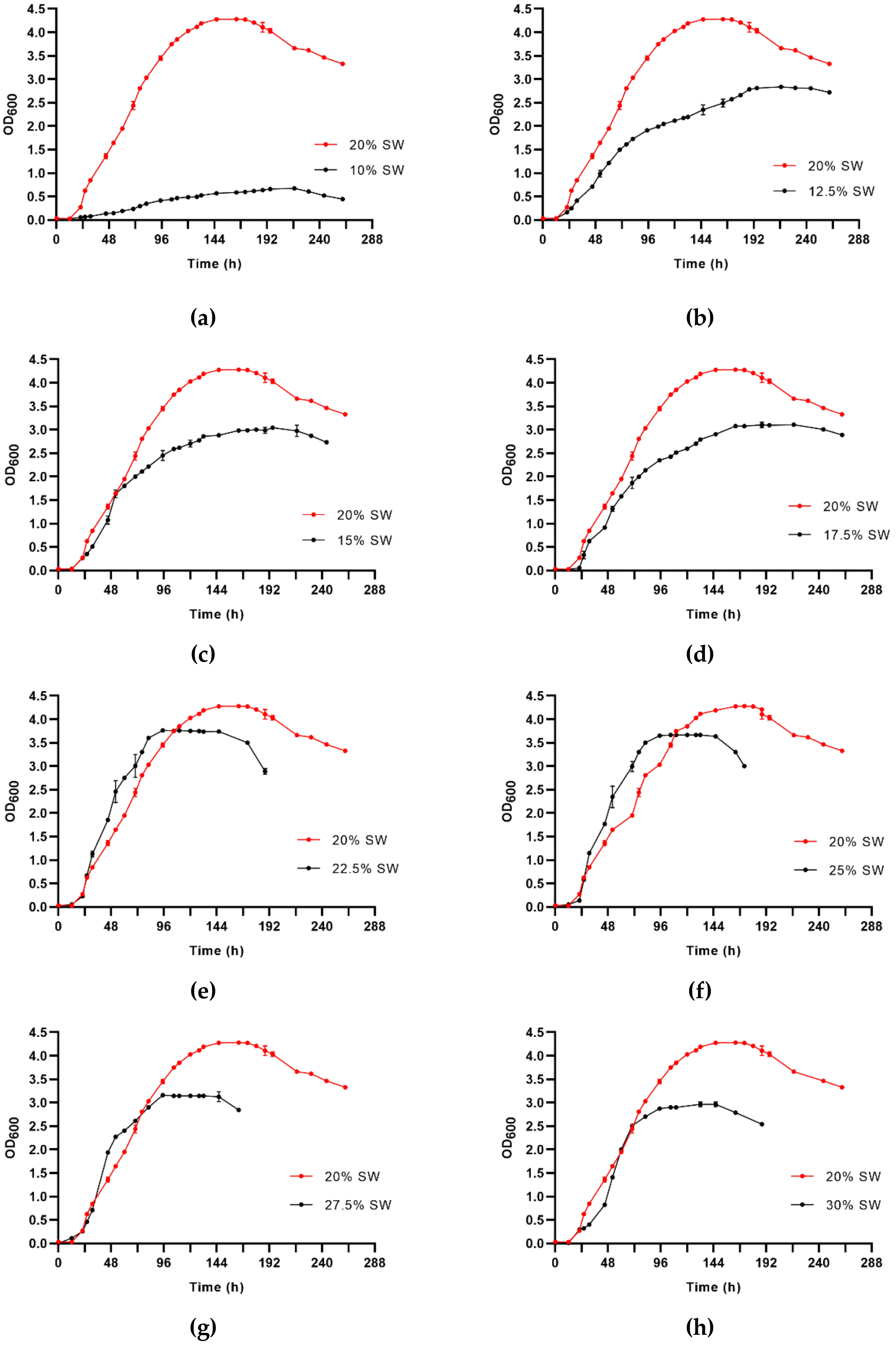

OD600
Time (h)
20% SW
10% SW
(a)
20% SW
12.5% SW
(b)
20% SW
15% SW
(c)
20% SW
17.5% SW
(d)
20% SW
22.5% SW
(e)
20% SW
25% SW
(f)
20% SW
27.5% SW
(g)
20% SW
30% SW
(h)

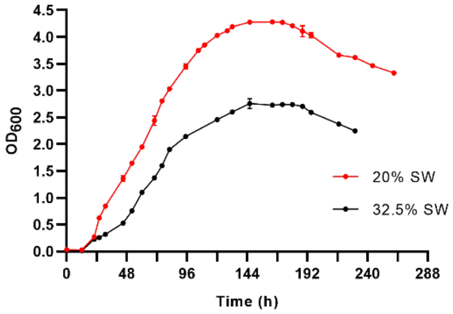

(i)

Figure S1. Growth of *Hfx. mediterranei* R4 (●) under standard culture conditions (20% SW, 20 mM NH_4Cl, 50 mM MOPS, 0.03 mM $FeCl_3$, 7.5 mM $CaCl_2$, 22.75 mM glucose. The pH was adjusted to 7.3 and grown at 42 °C. No metal addition.) and (●) under salt stress conditions: a) 10% SW; b) 12.5% SW; c) 15% SW; d) 17.5% SW; e) 22.5% SW; f) 25% SW; g) 27.5% SW; h) 30% SW; i) 32.5% SW.

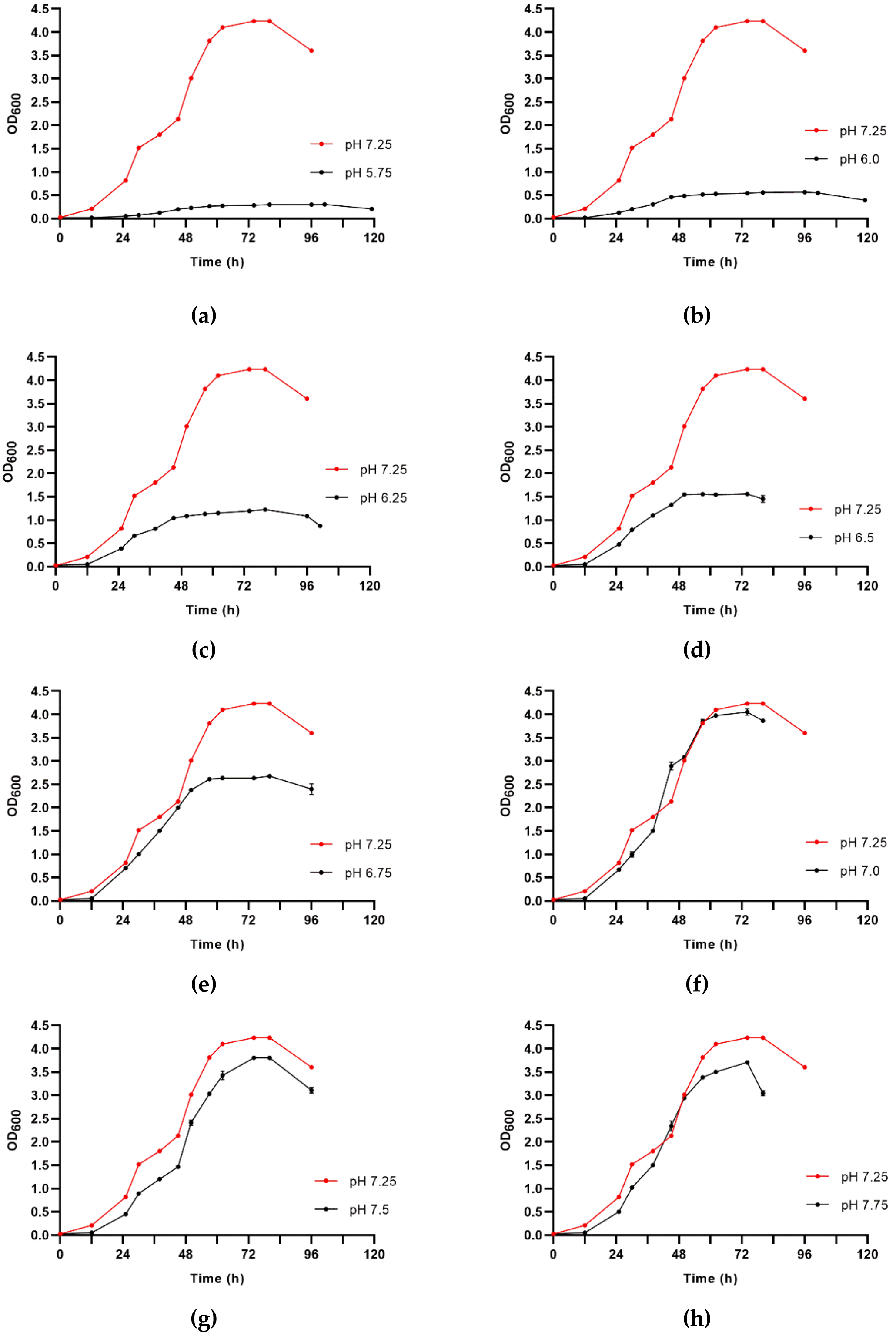

OD600
Time (h)
pH 7.25
pH 5.75
pH 6.0
pH 6.25
pH 6.5
pH 6.75
pH 7.0
pH 7.5
pH 7.75

(a) (b) (c) (d) (e) (f) (g) (h)

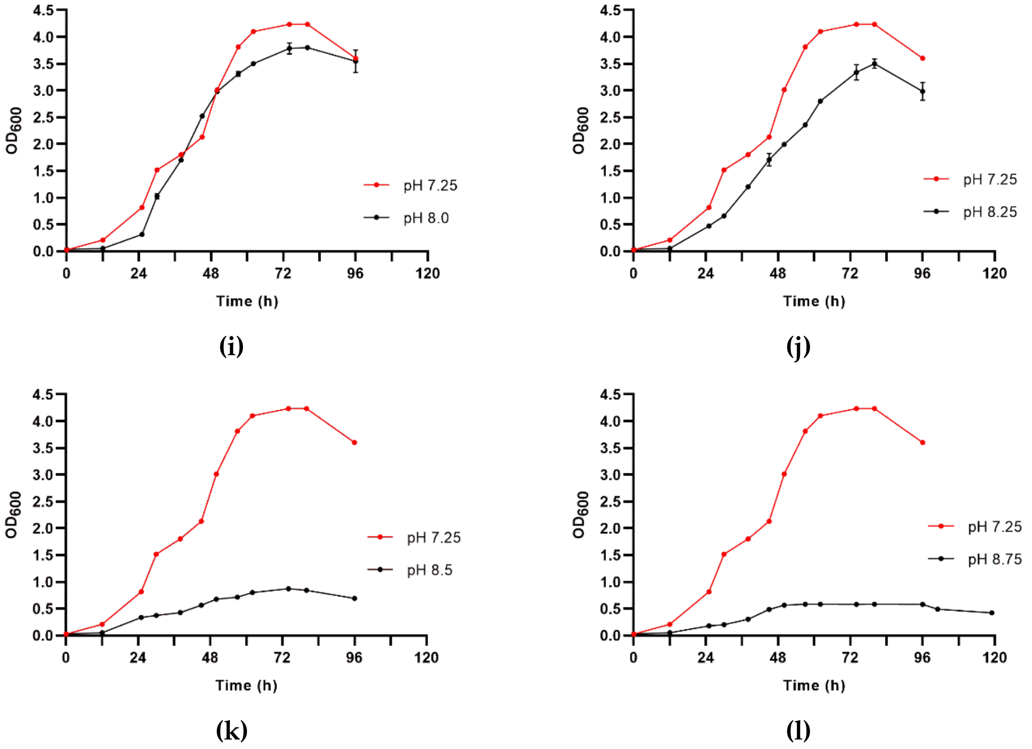

Figure S2. Growth of *Hfx. mediterranei* R4 (●) under standard culture conditions (20% SW, 20 mM NH_4Cl, 50 mM MOPS, 0.03 mM $FeCl_3$, 7.5 mM $CaCl_2$, 22.75 mM glucose. The pH was adjusted to 7.3 and grown at 42 °C. No metal addition.) and (●) under pH stress conditions: a) 5.75; b) 6.0; c) 6.25; d) 6.5; e) 6.75; f) 7.0; g) 7.5; h) 7.75; i) 8.0; j) 8.25; k) 8.5; l) 8.75.

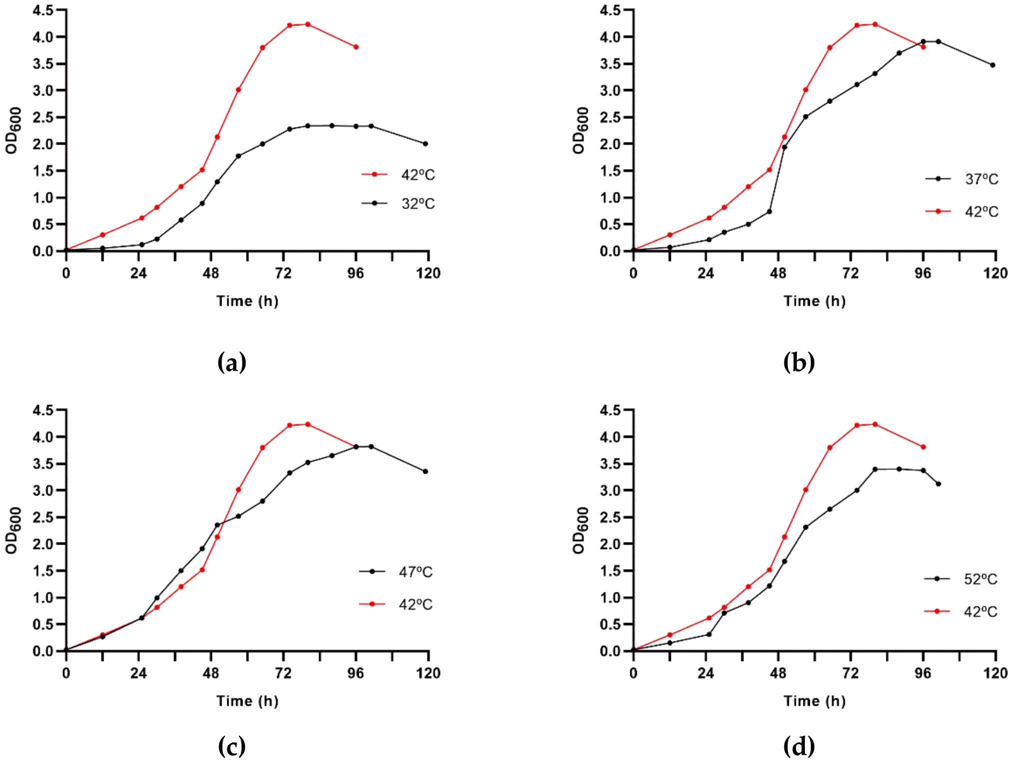

Figure S3. Growth of *Hfx. mediterranei* R4 (●) under standard culture conditions (20% SW, 20 mM NH_4Cl, 50 mM MOPS, 0.03 mM $FeCl_3$, 7.5 mM $CaCl_2$, 22.75 mM glucose. The pH was adjusted to 7.3 and grown at 42 °C. No metal addition.) and (●) under temperature stress conditions: a) 32 °C; b) 37°C; c) 47 °C; d) 52 °C.

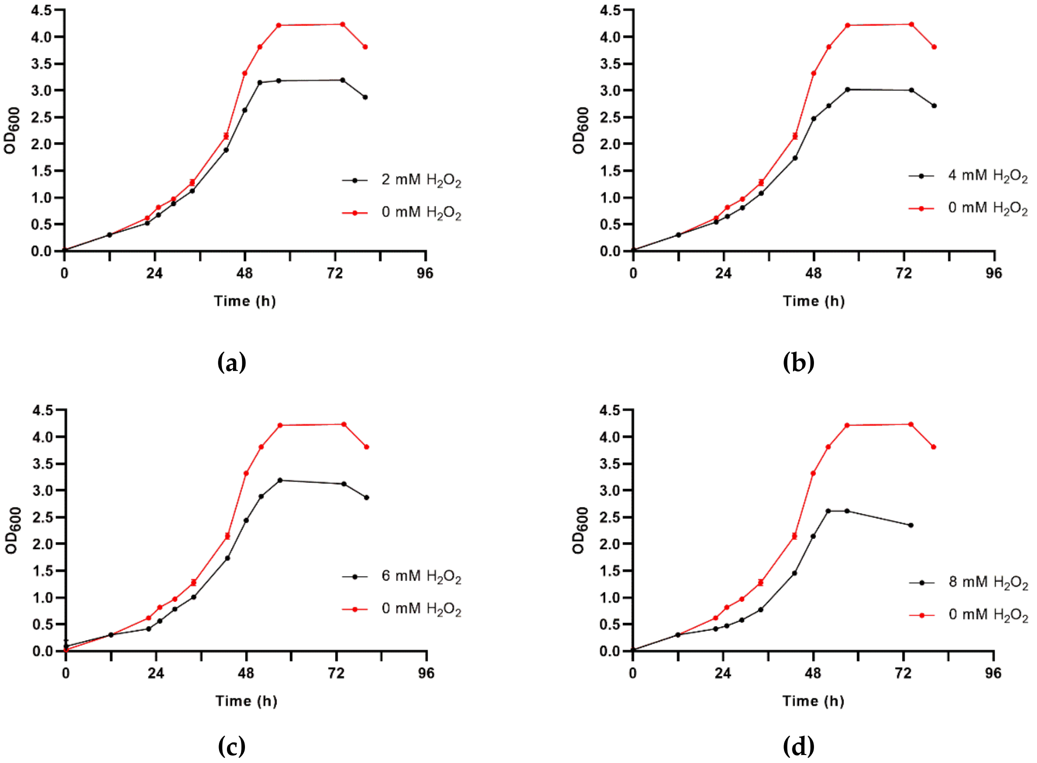

Figure S4. Growth of *Hfx. mediterranei* R4 (●) under standard culture conditions (20% SW, 20 mM NH_4Cl, 50 mM MOPS, 0.03 mM $FeCl_3$, 7.5 mM $CaCl_2$, 22.75 mM glucose. The pH was adjusted to 7.3 and grown at 42 °C. No metal addtion.) and (●) under oxidative stress conditions induced by: a) 2 mM; b) 4 mM; c) 6 mM; d) 8 mM.

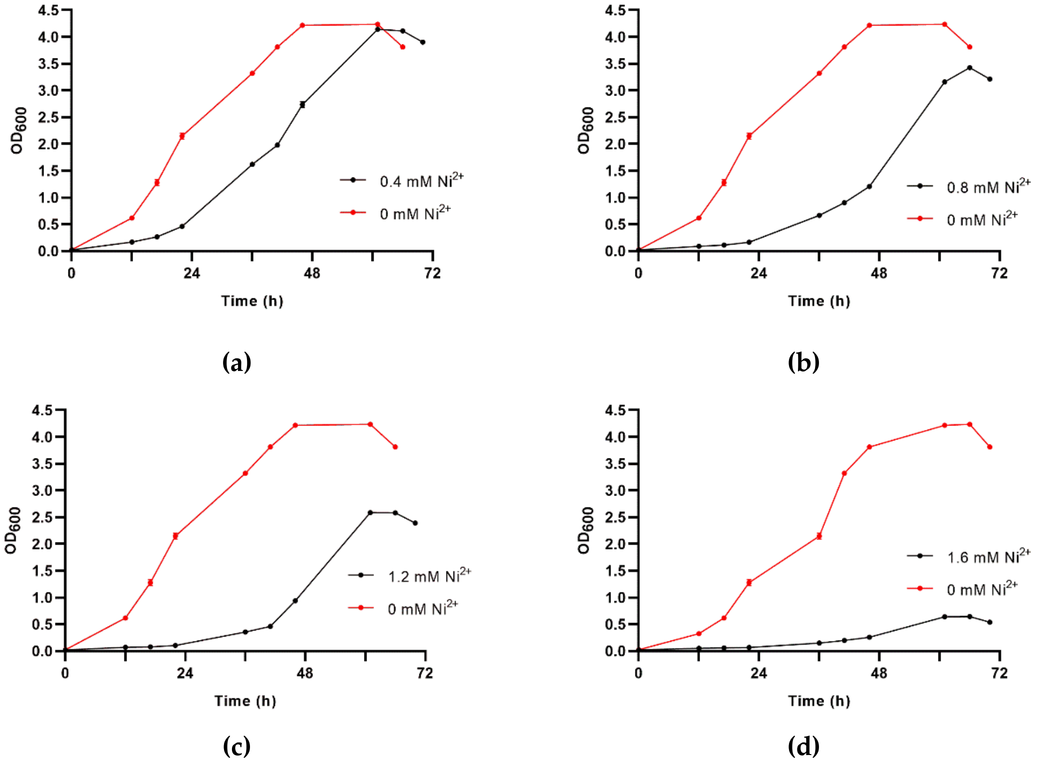

Figure S5. Growth of *Hfx. mediterranei* R4 (●) under standard culture conditions (20% SW, 20 mM NH_4Cl, 50 mM MOPS, 0.03 mM $FeCl_3$, 7.5 mM $CaCl_2$, 22.75 mM glucose. The pH was adjusted to 7.3 and grown at 42 °C. No metal addtion) and (●) under metal stress conditions induced by nickel (Ni^{2+}): a) 0.4 mM; b) 0.8 mM; c) 1.2 mM; d) 1.6 mM.

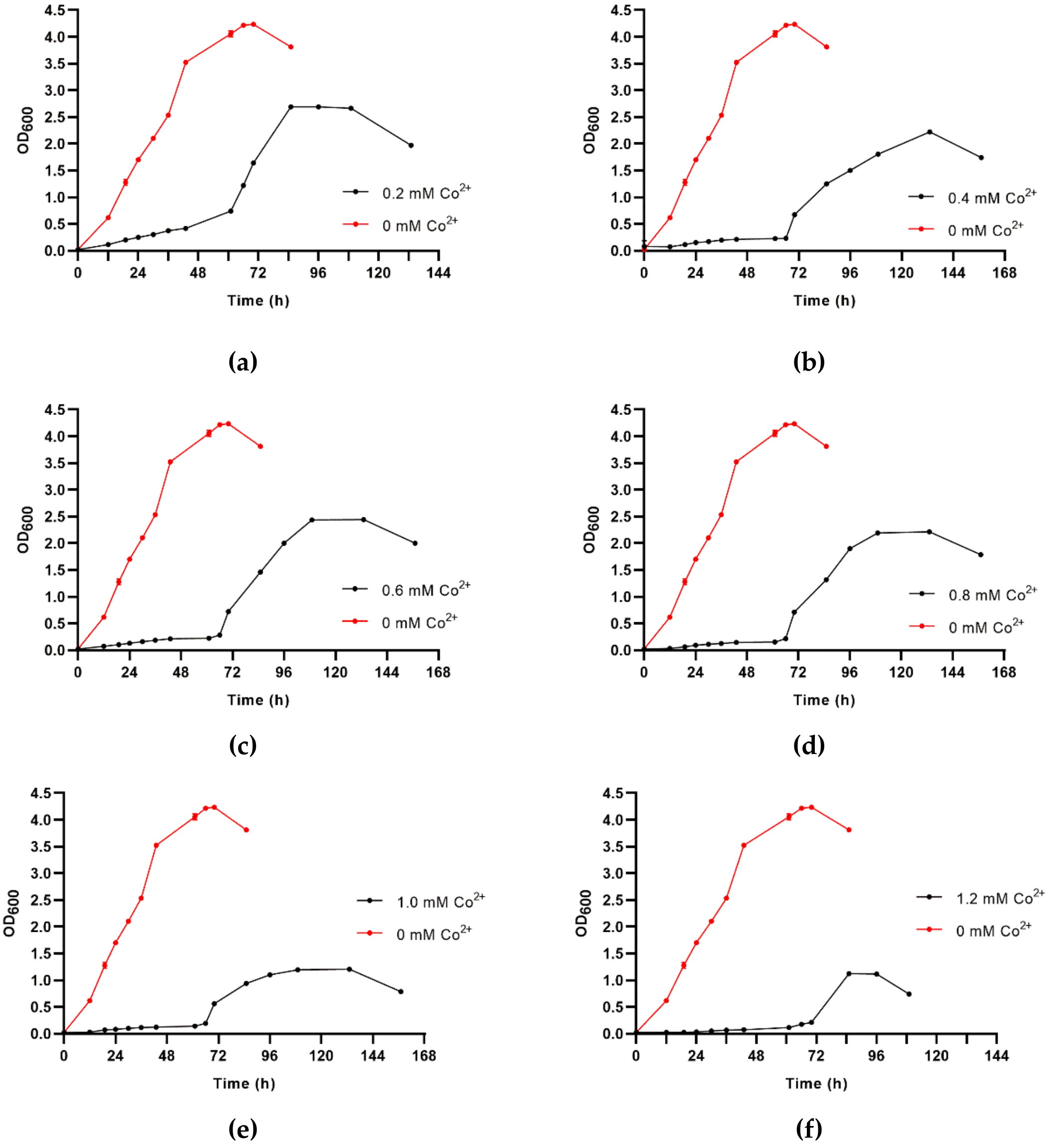

Figure S6. Growth of *Hfx. mediterranei* R4 (●) under standard culture conditions (20% SW, 20 mM NH_4Cl, 50 mM MOPS, 0.03 mM $FeCl_3$, 7.5 mM $CaCl_2$, 22.75 mM glucose. The pH was adjusted to 7.3 and grown at 42 °C. No metal addtion.) and (●) under metal stress conditions induced by cobalt (Co^{2+}): a) 0.2 mM; b) 0.4 mM; c) 0.6 mM; d) 0.8 mM; e) 1.0 mM; f) 1.2 mM.

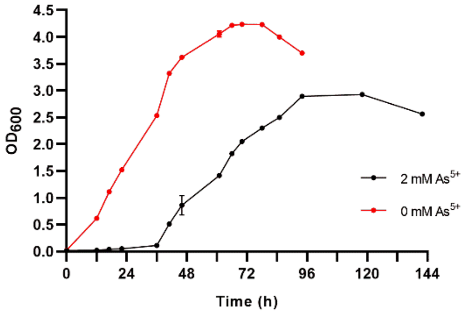

(a)

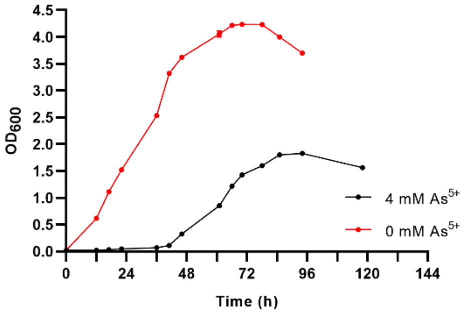

(b)

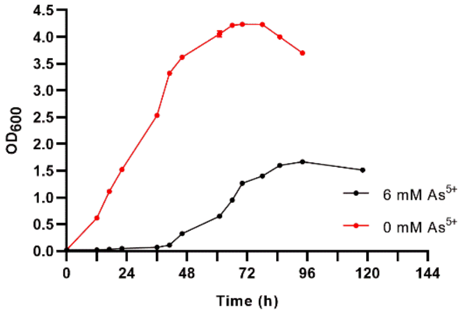

(c)

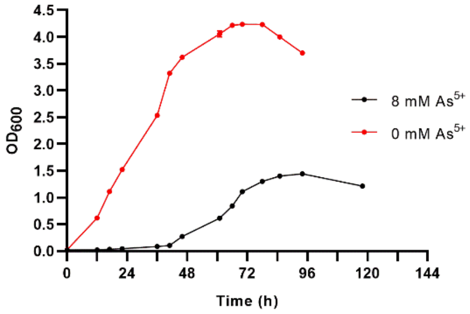

(d)

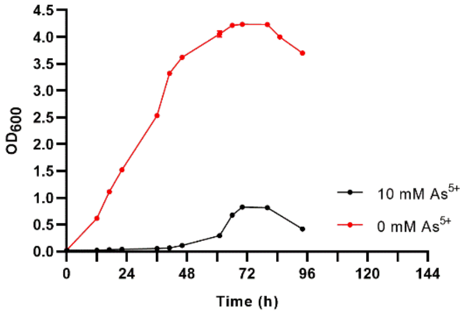

(e)

Figure S7. Growth of *Hfx. mediterranei* R4 (●) under standard culture conditions (20% SW, 20 mM NH_4Cl, 50 mM MOPS, 0.03 mM $FeCl_3$, 7.5 mM $CaCl_2$, 22.75 mM glucose. The pH was adjusted to 7.3 and grown at 42 °C. No metal addtion.) and (●) under metal stress conditions induced by arsenic (As^{5+}): a) 2 mM; b) 4 mM; c) 6 mM; d) 8 mM; e) 10 mM.

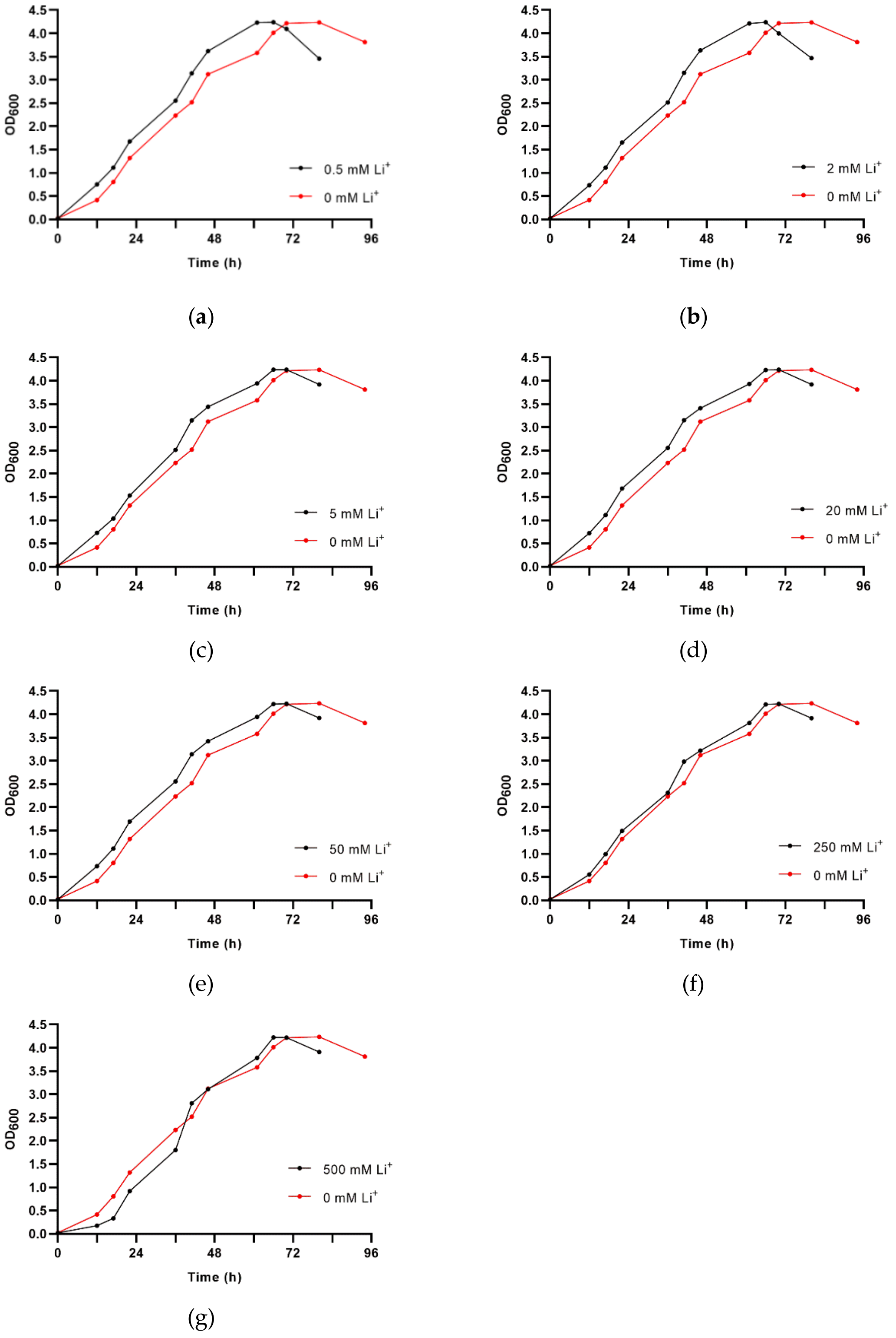

Figure S8. Growth of *Hfx. mediterranei* R4 (●) under standard culture conditions (20% SW, 20 mM NH_4Cl, 50 mM MOPS, 0.03 mM $FeCl_3$, 7.5 mM $CaCl_2$, 22.75 mM glucose. The pH was adjusted to 7.3 and grown at 42 °C. No metal addtion.) and (●) under metal stress conditions induced by lithium (Li^+): a) 0.5 mM; b) 2 mM; c) 5 mM; d) 20 mM; e) 50 mM; f) 250 mM; g) 500 mM.
